# Ethnographic process evaluation in primary care: explaining the complexity of implementation

**DOI:** 10.1186/s12913-014-0607-0

**Published:** 2014-12-05

**Authors:** Arwen E Bunce, Rachel Gold, James V Davis, Carmit K McMullen, Victoria Jaworski, MaryBeth Mercer, Christine Nelson

**Affiliations:** Kaiser Permanente Center for Health Research, 3800 N. Interstate Ave., Portland, OR 97227 USA; OCHIN, Inc., 1881 SW Naito Parkway, Portland, OR 97201 USA; Multnomah County Health Department, 426 SW Stark St., Portland, OR 97204 USA; Virginia Garcia Memorial Health Center, PO Box 568, Cornelius, OR 97113 USA

**Keywords:** Ethnography, Qualitative methods, Mixed methods, Clinical informatics, Primary care

## Abstract

**Background:**

The recent growth of implementation research in care delivery systems has led to a renewed interest in methodological approaches that deliver not only intervention outcome data but also deep understanding of the complex dynamics underlying the implementation process. We suggest that an ethnographic approach to process evaluation, when informed by and integrated with quantitative data, can provide this nuanced insight into intervention outcomes. The specific methods used in such ethnographic process evaluations are rarely presented in detail; our objective is to stimulate a conversation around the successes and challenges of specific data collection methods in health care settings. We use the example of a translational clinical trial among 11 community clinics in Portland, OR that are implementing an evidence-based, health-information technology (HIT)-based intervention focused on patients with diabetes.

**Discussion:**

Our ethnographic process evaluation employed weekly diaries by clinic-based study employees, observation, informal and formal interviews, document review, surveys, and group discussions to identify barriers and facilitators to implementation success, provide insight into the quantitative study outcomes, and uncover lessons potentially transferable to other implementation projects. These methods captured the depth and breadth of factors contributing to intervention uptake, while minimizing disruption to clinic work and supporting mid-stream shifts in implementation strategies. A major challenge is the amount of dedicated researcher time required.

**Summary:**

The deep understanding of the ‘how’ and ‘why’ behind intervention outcomes that can be gained through an ethnographic approach improves the credibility and transferability of study findings. We encourage others to share their own experiences with ethnography in implementation evaluation and health services research, and to consider adapting the methods and tools described here for their own research.

**Electronic supplementary material:**

The online version of this article (doi:10.1186/s12913-014-0607-0) contains supplementary material, which is available to authorized users.

## Background

Health services researchers are paying increasing attention to the value of using a mixed methods approach to enrich understanding of the complexities of health care delivery and practice transformation [1-3]. Recent literature explores the integration of quantitative and qualitative methods [4] and emphasizes the need for rigorous, methodologically sound methods, as well as detailed and transparent reporting on qualitative *and* quantitative methodology [3,5-8]. In response, this paper describes an ethnographic approach to process evaluation in the context of a mixed methods convergent design within an intervention framework [9]. We present in detail the qualitative methods used to study the translation of a primary care health information technology (HIT)-based quality improvement intervention from an integrated care setting to community clinics. While others have called for the use of ethnography in studying healthcare [10,11] and HIT [12,13], the specific methods used in such process evaluations are rarely presented in detail.

This paper demonstrates that an ethnographic approach to evaluating implementation encourages reflection, flexibility and openness to new ideas – and, when informed by and integrated with quantitative data, results in a rich, nuanced picture of the implementation process. Our goal is twofold: a) to stimulate a conversation around the successes and challenges of specific data collection methods in practice settings, and b) to offer an example of accessible, pragmatic qualitative methods that can be modified and adopted by health services researchers seeking to explain implementation complexity in primary care settings. We focus here on the qualitative data collection methods (weekly diaries by clinic employees; observation; informal and formal interviews; document review; surveys and group discussions). Future manuscripts will describe our integration of quantitative and qualitative methods and data, and present our analyses and study results.

The ALL (Aspirin, Lisinopril, Lovastatin) Initiative is a population-level intervention developed and launched nationally by Kaiser Permanente (KP). It uses electronic health record (EHR)-based tools to increase the percentage of patients with diabetes who are appropriately prescribed evidence-based cardioprotective medications. A 2009 KP internal review estimated that implementing the ALL Initiative led to a 60% reduction in cardiovascular events among targeted patients [14]. Our current NHLBI-funded study investigates the feasibility of adapting this intervention for implementation in 11 community health centers (CHCs) in Portland, OR. The study evaluates this cross-setting translation using both quantitative measures (percent of patients with appropriate prescriptions each month; rates of provider use of the HIT tools) and predominantly qualitative process and contextual variables. The quantitative data show the ‘what’ – the results of implementing this intervention – while the qualitative process evaluation methods described here delve into the ‘how’ and ‘why’ behind these results. We received approval from the Kaiser Permanente NW Institutional Review Board to obtain verbal consent for all data collection related to this process evaluation.

Process evaluations typically focus on fidelity – the extent to which a program was implemented as intended in a new setting [15-17]. This emphasis can lead researchers to overlook the myriad, seemingly mundane details that may affect an intervention’s ultimate success, such as: who did what when; how people felt and talked about the intervention; how relationships, hierarchies, and workflows changed; and the resistance, compromises, and workarounds that arose when a particular intervention was introduced in a specific setting and time [11,18-20]. Such details often are crucial to understanding the intervention’s impact, and vital to guiding context-specific refinement of the implementation approach and the intervention itself.

Ethnographic methods are explicitly intended to collect the kind of detailed data that fidelity-focused process evaluations rarely address. Ethnography uses naturalistic observation and face-to-face interaction [21], i.e., what is seen, heard and experienced [22], to illuminate the dynamics underlying intervention outcomes. These dynamics unfold within a specific context – in this case, daily life in a CHC – that shapes how clinic staff perceive the intervention. Contextual factors are key to understanding how participants understand and react to an intervention [11,18]. Intervention outcomes cannot be understood without considering variables such as practice setting, culture and history; national, state, local and organizational policies; community norms and resources; payment and incentive systems; patient characteristics; and the culture around monitoring and evaluation [23]. Contextual factors are best assessed using methods that engage diverse perspectives, consider multiple levels, consider changes over time, look at both formal and informal systems and culture, and assess interactions between contextual factors and process and outcomes measures [24], all of which are hallmarks of ethnography.

Ethnography’s overarching goal (to understand an intervention and its impact from the participants’ perspective [25]) dovetails with that of process evaluation within implementation science (to study what mediates or moderates intervention effects [26]). An ethnographic approach to process evaluation emphasizes placing the intervention in its historical and social context, “being there” to document the process as it unfolds and as interpreted by its participants, openness to unanticipated consequences [20], and illumination of multiple, complex, and competing perspectives [18]. Thus, it can uniquely inform an important but often neglected component of process evaluations: *What is happening, and why* [20].

Answering this question is particularly important when evaluating the implementation of HIT-based interventions. Such interventions’ value is often assessed via primarily technical questions, e.g., Were the tools used or not? How can the tools work faster/more accurately/better fit the workflow?. These approaches ignore the complexity of the real-world settings in which HIT is used. The success of *any* intervention depends on variables involving power structures, social control, meaning, values, emotions, and relationships, all of which exist in the context of specific historical, social, and cultural settings [12,13,25,27,28]. These considerations certainly apply to HIT-based interventions, which are embedded in complex sociotechnical interactions that form the daily work of health care [12,23,29-31]. Thus, the study of adoption and use of *HIT-related interventions* requires consideration of the complexities of *both* health care and information technology, and how the two interact. Ethnography acknowledges the ambiguity, unpredictability, and diverse perspectives that comprise implementation in practice settings. An ethnographic approach to HIT-related process evaluations could, therefore, provide a necessary counterpoint to the potentially reductionist “single story” [32] view of why an intervention succeeds or fails in a particular setting.

## Discussion

### Methods

We modified traditional ethnographic data collection methods, such as key informant and in-depth interviews, focus groups, naturalistic observation, journals, surveys and collection of artifacts [33], to study the process of an evidence-based primary care intervention implementation in CHCs. We sought to identify barriers and facilitators to implementation success, provide insight into the quantitative study outcomes, and uncover lessons potentially transferable to other implementation projects [10,34] – and to do so in a methodologically rigorous manner acceptable to busy primary care clinicians. Modifications were necessary to meet the expectations of clinic leadership, who in exchange for facilitating research access hoped to gain timely, actionable information that could be used to improve staff morale and clinical care. To reduce clinic burden we used less intrusive methods (weekly diaries by site coordinators, short surveys, document review, workflow observation) as our primary form of data collection, and limited methods that require clinician time off the floor (interviews, group discussions) to filling in details and challenging or corroborating findings. Table 1 provides additional details on the adapted methods.

**Table 1:** **Summary of ethnographic methods used to study the implementation of an evidence-based primary care intervention in CHCs**

	**Method**	**Previous research**	**Innovation**	**Detail**
*Used to discover key themes and patterns*	Site Coordinators (SCs)	- Hired from outside the organization	- Hired from within the organization	Nurses and quality improvement specialists, 2.5 FTE between 4 SCs; training in interview methodology + ongoing informal methods training
		- Assist with implementation (practice facilitation)	- Play an essential role in data collection, analysis/interpretation (as well as practice facilitation)	
	Diary entries completed by SCs	- Structured format with set open-ended questions	- Unstructured format, no set questions	Submitted weekly; email reminders; qualitative lead collates each month’s entries by site, inserts questions & comments & returns to SC for clarification; relevant practices and ideas shared with team
		- Rolled out with no training	- Training that emphasizes rich description and the value of the diarists’ own knowledge and insight	
		- One way flow of information from diarist to researcher	- Feedback loops between diarist and researcher regarding diary content	
	ECCO (Episodic Communication Channels in Organization) surveys	- Measure spread of multiple messages to capture communication channels in a given organization	- Snapshot of success of communication strategies by role related to a single intervention	Anonymous 2-page paper survey conducted 1 year post-implementation at 5 clinics; completed by 64 individuals (15 PCPs*, 12 RNs**, 17 MAs***, 20 other (pharmacy, panel managers, etc.)
	Document review	- Collect relevant clinic documents to gain insight into organizational culture and context	- Same; also collect relevant email communications to document team interactions within and across organizations	SCs forward clinic documents to qualitative team, with explanations placing them in context. As of study year 4, have 191 such documents.
	Observation	- Observation of clinical workflow	- Same; also view team meetings as ethnographic encounters worthy of study	Informal observation in clinics and at team meetings throughout study; formal half day shadowing of 1–2 clinicians per clinic. Study year 3 & 4.
*Used to challenge or corroborate findings; fill in detail*	Group Discussion	- Scheduled as stand-alone discussions with pre-selected participants	- Integrated into regular clinic meetings	1-2 group discussions per clinic, with PCPs, RNs, MAs and panel managers (divided by role); Discussions last 0.5-1 hr; Recorded & transcribed. Study years 3–5.
		- Emphasis on confidentiality; no clinic leadership present	- Usually co-conducted with clinic leadership	
		- Principal method of data collection	- Used to fill in knowledge gaps and test researcher understandings	
	Interviews	- Conducted by single outside researcher	- Co-conducted by outside researcher and site coordinator	4-6 interviews per clinic
				(2–3 PCPs, 0–1 RNs, 0-2 management) Recorded & transcribed. Study years 3–5.
		- 0.5-1 hr in length	- 20 mins in length (1 patient encounter)	
		- Principal method of data collection	- Used to fill in knowledge gaps and test researcher understandings	
*Human subjects protection*	Informed consent	- Signed	- Verbal	Ethics board considered the process evaluation low risk.

*Primary care provider; **Registered nurse; ***Medical assistant.

As of Year 4 of this five-year study, we have collected over 300 data documents (field notes, transcripts, etc.) through the methods detailed below. As is standard in qualitative research, data analysis is an ongoing, recursive process of reading, discussing and reflecting on the data as it is collected [35,36]. Subsequent data collection is customized based on emerging understandings and identification of key knowledge gaps. Although the analysis of ethnographic data can take various forms, it is essentially a dynamic process of organizing, describing, interpreting and legitimating raw (often text) data in order to make sense of the information [37-39]. Inconsistencies, tensions and ambiguities in the data are explored and reported, as they illuminate the often messy complexity of real-world settings [18]. A typical analysis process involves recursive cycles of immersion in the data, identifying and applying codes (labels assigned to text segments to identify and categorize emergent or previously defined key concepts [36]), reflecting on and discussing developing understandings, and collecting additional data as necessary [35,36,38]. This process is repeated until saturation, or the point at which no new information or themes are observed in the data [40] – and until “reportable interpretations” ([35], pg. 180) are reached. While analysis is ongoing and we continue to fine-tune our methods as circumstances demand, this ethnographic approach to process evaluation is yielding in-depth, nuanced data from multiple perspectives that, in conjunction with quantitative outcome data, captures the complexity of the implementation process and the factors affecting implementation success. We describe the qualitative data collection methods used, and discuss the successes and challenges of each.

## Results

### Verbal consent

As the intervention was a publicly documented organizational activity, the ethics board considered the process evaluation to be low risk and approved the use of verbal consent. This suited local practice and expectations (clinicians are accustomed to being asked for feedback on quality improvement initiatives) and allowed for flexibility when collecting data under time-limited circumstances.

### Site coordinators

The process evaluation is led by two study team researchers, with substantial assistance from four ‘site coordinators’ from the study CHCs. The site coordinators were hired with study funds to oversee the intervention’s implementation, and to link the clinics and the research team. Each CHC group chose established employees (nurses and quality improvement specialists) to fill this role; this decision proved instrumental to the research process. We heard repeatedly that site coordinators’ previously established relationships in each organization were key to the initial and ongoing willingness of staff to consider making these changes to clinical practice, and to sharing their experiences. Site coordinators can also ‘vouch’ for the research team. As one provider noted to a site coordinator: “It’s a good thing that you are the one doing this ALL study, because we love you. Anyone else, I would have to hate them.”

As insiders immersed in the daily life of the organization, the site coordinators help the research team understand decisions and actions in context; share (as appropriate) information from relevant meetings, initiatives and conversations; provide an ‘insider’ view of the clinic and organizational culture; and help interpret study data in historical, interpersonal, and workflow context. They help tailor our data collection strategy to the specific setting, e.g., How many people can we pull out of clinic to interview? How do we pitch our work to management and busy clinicians?. This filtering and interpretation is a vital ‘insider’ role; it would be next to impossible for someone from outside the organization to uncover and/or understand the interconnected pieces and local history that contribute to intervention uptake or rejection.

Site coordinators were oriented to ethnographic data collection during a two-hour in-person training that focused on: 1) the goal of ethnographic data collection in implementation research (to learn how things look and feel from the perspective of those impacted); and 2) asking good questions and learning to listen (establish rapport, maintain neutrality, provide a framework for respondents to express things in their own terms, probe, question assumptions). The training also included some within-group interviewing exercises and feedback. With practice and ongoing informal discussions with each other and the qualitative study team, the site coordinators have proven particularly adept at capturing staff feedback on the intervention’s HIT tools and implementation process. Details of such conversations are recorded in weekly diaries and provide both great insight into specific issues and a starting point for further exploration of unexpected findings.

### Weekly diaries

The process behind using weekly diary entries to effectively gain this grounded insight into barriers and facilitators to implementation required some trial and error. Our original diary form was quite structured. It consisted of five text boxes, one for each intervention “tool” (best practice alert in the EHR, outreach roster, etc.) and asked the site coordinators to record feedback on each, specifying for each comment the specific tool, staff position and clinic. No other guidance was given. While this format identified some helpful clinic-level questions and concerns, the feedback was fairly limited in scope, and often the diaries were returned with “nothing to report”. Consequently, in the second year of the study we restructured the diary form and process. The form was revised to simply say “Please include anything you think might help us understand barriers and facilitators to [the] implementation”, with a few reminders and a list of potential topics followed by an empty text box [see Additional file 1]. The new form was introduced at a two hour in-person training led by the qualitative team. The training emphasized:

- *Why* we were asking for this information - daily routine and challenges crucial to understanding the process of implementation [41]; space for reasoning and reflection; interactive learning environment;
- *What* information we sought - things you have heard, observed, done, think, or know;
- *How* to write about it - rich detailed descriptions; differentiate between description and interpretation (tell the story);
- The *value* of the site coordinators’ own knowledge and insights.

The response was remarkable; under the new format diary entries range from one paragraph to three pages, and include descriptions of conversations, de-identified excerpts from patient charts, and observations and thoughts related to intervention implementation that provide detailed insight into the ongoing complexities of the implementation process. Once a month the study’s ethnographer collates each site’s entries from the previous month, inserts comments and follow-up questions, and returns it to the site coordinator for clarification and discussion. Relevant questions, ideas or practices are then shared across sites and with the larger team, and discussed at monthly meetings of the site coordinators and qualitative researchers. Preliminary analyses of this data pointed to the importance of issues related to trust (of evidence, of management, of the site coordinator, of the EHR and EHR-based tools); provider views on appropriate clinical prioritization; and how the presentation of study data/performance metrics affect intervention uptake. These insights led to clinic-initiated changes in the content and format of communication around the intervention. Relevant topics were also subsequently explored in greater detail through targeted questions in informal and formal interviews.

One key to successfully using diaries to collect ethnographic data on intervention implementation may be active engagement with the ‘diarists’ about the content of the entries. This is a different style and content of writing than most people are accustomed to, perhaps particularly those in the clinical world; it took time for the site coordinators to become used to looking for the “stories” and to feel comfortable sharing their views. The diaries are intended to be a conversation between the ethnographer and site coordinator. In the first few months after restructuring the diary process our ethnographer often had to ask for additional details and interpretation, i.e.: What did the clinic staff actually say? What do you think it meant? What is the history to that interaction? As time went on and the methodology became more clear to all involved, the (written) conversation became more two-sided, as the ethnographer and site coordinators discussed: What might this mean? Is this a pattern we might see across sites? How can we find out more? In sum, rather than the typical model in which the writer sends information in a one-way stream, site coordinators have been actively involved in shaping the data collection, interpreting its meaning, and figuring out where to take it next. They can see how the information they provide is used to gain an in-depth view of the day-to-day realities of intervention uptake.

### ECCO survey

We created an anonymous survey based on Episodic Communication Channels in Organization (ECCO) methods to assess the spread and accuracy of information about the ALL intervention across the study clinics and their staff one year post implementation [42]. While ECCO surveys are typically used to measure the spread of multiple messages in an organization to capture overall communication channels [43], we designed ours to assess the success of communication strategies used to support practice change related to uptake of a single intervention. The short (2–3 minute) survey asked which of the main intervention messages respondents had heard, from whom, in what context, and when [see Additional file 2]. Site coordinators distributed the paper survey to a cross-section of clinic staff at each site, including primary care providers, nurses, medical assistants, pharmacists, team assistants, patient care coordinators and clinic management. The survey required few resources or staff time, and provided some intriguing results about the impact of clinic and team dynamics and staff roles on intervention-related knowledge and uptake. Differences in intervention knowledge were more apparent between individual clinics than between organizations, for instance, possibly indicating that internal team and clinic dynamics have more influence than organizational leadership on intervention-related communication. We also learned that some providers were confused about (or perhaps resistant to) prescribing these cardioprotective medications to younger adults (age 18–54), and that almost a quarter of respondents did not know that the intervention’s care recommendations were evidence-based and would continue once the study was complete. Clinic leadership received a summary of the survey findings, and some revised their intervention-related messaging to directly address knowledge gaps or concerns related to the evidence base.

### Document review

To capture the nuances of team interactions and gain insight into clinic and organizational culture and context, we are collecting relevant communications and documents, as appropriate. These documents include: email strings among team members, on subjects such as the potential impact of new EHR features on our study tools; in-house newsletters; diabetes flow charts; presentations; announcements; meeting notes; and site-specific standardized workflows, with explanations by the site coordinators as necessary. These documents help place our intervention implementation within the larger context of clinic life. The cross-site team communications highlight the different priorities and capacities of the organizations involved in the study and the negotiation and compromises necessary for successful implementation – as well as ‘pain points’ that threaten to derail the process. The struggle to achieve inter-organizational consensus regarding clinical definitions (e.g., how to define hypertension for the purpose of this intervention) and workflows and yet still allow a reasonable degree of clinic-level customization and autonomy, for instance, comes through clearly in these documents. The clinic documents also reveal the constantly changing landscape of life in CHCs as management, providers and staff struggle to provide the best possible care to their patients in the face of budget constraints and a shifting policy, regulatory and financial landscape. The study spans the continuation of the Medicare and Medicaid EHR Incentive Programs (“meaningful use”) [44] and the introduction of the Affordable Care Act and Accountable Care Organizations (ACOs). We can trace changes in clinic workload (one organization was assigned over 4,000 new patients in less than four months), pharmacy formularies and copays that directly impact the ability of patients to obtain ACEI/ARBs and statins, and clinic staffing as it relates to the introduction of ACOs. Two of the three study organizations also participated in the Alternative Payment Methodology (APM) demonstration project [45], which allowed participating CHCs to shift from earning revenue based on the number of individual patients seen to a monthly payment based on the size and composition of their patient population – with significant implications for workflow and team-based care.

### Observation

Over the course of the study, qualitative team members have become familiar to clinic and study staff. We attend monthly on-site meetings with study-affiliated clinic staff, biweekly study team calls, intervention-related trainings, and other relevant meetings. We participate in discussions about the content, build and iterative adaptation of the intervention tools with our HIT and clinical team members, and listen as they debate HIT’s role in primary care workflows. When in the clinics for more formal data collection activities (meetings, trainings, interviews) we often take time to engage in informal conversations with and observe the workflow of available staff, focusing on staff interaction with the EHR and use (or non-use) of the intervention’s alert/reminder and reporting/panel management tools. We document these interactions, and our interpretations of them, in detailed narrative field notes.

In addition, we conduct more structured observations. We shadow teams for a half day at each of the 11 study clinics. At each clinic, the site coordinator identifies one or two providers with a cluster of diabetes appointments. Once an observation window is identified, the site coordinator requests provider permission for a research team member to observe team workflow during this time period including - with patient consent - patient encounters. During shadowing we try to observe all workflow elements including team meetings, provider/medical assistant pre-visit ‘scrubs’ or ‘huddles’ , patient rooming, and RN and provider sessions. Our goals are to gain a concrete understanding of the diabetes-related clinic workflows, to ground our analyses in the actual day-to-day work of the clinic, and to illuminate potentially important differences in workflow by organization and individual clinic. We also find that this type of direct observation can deepen our understanding of issues identified through other forms of data collection. We knew, for instance, that the study-provided individual performance metrics were sometimes deemed incorrect by providers; shadowing of one doctor illustrated the multitude of reasons the data did not always accurately reflect the situation (patients taking family members’ medications, deliberate provider decisions based on individual understanding of the evidence and of their patients’ lives, etc.) and the implications this seeming disjunction between the data and the providers’ reality can have with regard to trust in the intervention itself.

### Group discussions

Instead of the traditional focus group format, with its emphasis on privacy and confidentiality [46], we chose to work within existing clinic meeting structures, and to ‘tag team’ with the site coordinator and clinical lead at each site. A typical group discussion takes place during a routine staff meeting. Given constraints on provider time this is often our only option, but it can be difficult to get on the meeting schedule, and we are sometimes bumped at the last minute. It can also be difficult to persuade clinic managers to dedicate an entire meeting to our study – we are sometimes given 20 minutes of a one hour meeting which, by the time other items are covered, dwindles to 10. When given an entire meeting, the clinical lead or site coordinator spends the first 20–30 minutes sharing study results and answering questions. The ethnographer then uses the remainder of the meeting for a guided discussion. By starting with a ‘refresher’ we can dispense with the usual warm-up questions and launch straight into in-depth discussions of the most relevant issues. We can also refine our questions based on the content of the discussion in the first half of the meeting – we transition from refresher to group discussion as seamlessly as possible – to increase the relevance to participants and encourage spirited interaction. During the first part of one group discussion, for instance, the providers asked the clinical lead for guidance on study-specific outreach; the ethnographer then used that conversation as a springboard to a lively discussion on patient access issues. With consent, we record the entire discussion, including the first half led by clinic staff. Clinic staff are informed of the format at the beginning of the meeting, given study fact sheets, and asked if there were any objections. Although staff are told they are free to ask us to turn off the recorder at certain points, or to leave at any time, nobody has chosen to do so.

Through this approach, staff can learn more about the intervention and how their clinic was performing, the site PI and site coordinator can make timely changes to implementation strategies based on the feedback, and the research team gains valuable data for the process evaluation. Our initial concerns that participants would not feel free to express honest opinions with a member of clinic management in the room appear unfounded; participants told us that they *like* that their voices are heard directly by their clinic leaders instead of going into a research ‘black hole’. Nevertheless, we chose not to include any leadership figures when talking with employees in certain staff roles (medical assistants, panel managers, etc.) as we suspect they would be less comfortable speaking freely in the presence of clinic management. This collaborative approach may not work as well in contexts in which employment is more precarious or organizational culture inhibits open dialogue.

### Interviews

We use formal semi-structured interviews to fill in knowledge gaps identified after preliminary analysis of data gathered via less intrusive means (observation, document review, etc.), and to capture thoughts and opinions that participants may not be comfortable sharing in a more public setting. We interview clinic staff with diverse opinions about the ALL intervention – those who find it helpful, those who are resistant to HIT in general or this intervention in particular, and those with especially strong feelings, positive and negative, about specific elements of the intervention [47]. These interviewees are identified during prior data collection, by the site coordinators, and through review of quantitative study data (guideline-based high and low prescribers of the targeted medications). We make a point to occasionally interview members of the same team to explore the role of team communication on intervention uptake. Provider interviews are the most difficult to schedule: the study CHCs asked us to limit provider interviews to two to three 20-minute interviews per clinic, the length of a single patient encounter. At their request, clinics can charge the study for provider time off the floor. In most cases, we aim to conduct four to six interviews per clinic, focusing on providers, nurses and clinic management. Data from medical assistants and panel managers are obtained during observation, informal interviews and group discussions.

The most productive formal interviews are those conducted jointly by an ethnographer and site coordinator. Their complementary backgrounds and perspectives, and the balance of insider/outsider status, yields fruitful conversations [48]. The ‘outsider’ ethnographer is positioned to integrate and synthesize information across study sites and identify emergent patterns and notable outliers. The ‘insider’ site coordinator can pose questions based on intimate knowledge of clinic culture, workflow or team composition. In one exchange during a provider interview, for example, the ethnographer asked about the role of the team RN in alerting the provider to the potential need for an ACE/ARB or statin prescription as indicated by study logic; when the provider replied that this is not part of the RN workflow, the site coordinator (based on insider knowledge of current debates surrounding the RN role) asked under what circumstances she might be receptive - which triggered an interesting discussion of care team dynamics. In another interview, the ethnographer asked about the impact and use of study data reports; the site coordinator then used her detailed and contextual knowledge of outreach efforts at that clinic to ask focused questions that resulted in a nuanced description of the role of data in clinic outreach and patient care.

While site coordinators bring to the interviews a first-hand knowledge of the realities of the implementation process, it can be difficult as an insider to identify and push beyond shared assumptions. The ethnographer, by contrast, can ask the seemingly naïve questions that often illuminate previously unexplored thoughts and concepts. Respondents also try to explain their answers more thoroughly to an outsider. The ethnographer’s presence introduces some formality to the proceedings, which helps maintain focus and justify the use of a recorder. Site coordinators conducting interviews on their own sometimes feel awkward using a recorder, and the interviewees occasionally refuse it. One site coordinator explained, “I feel like [I] get the best information from staff when I am an “insider” who can relate to what they are saying. Turning on a recorder destroys that intimate bond…” While field notes from the peer-based informal interviews lead to important insights, analysis of the formal interviews benefits from the word-for-word transcripts possible only from a recording. A short post-interview debrief between the interviewers helps to fill in context or history underlying certain reactions, and enriches the ethnographer’s understanding of the interview data.

## Summary

The intersection of and interdependencies between technology and clinical practice are intricate and multifaceted, and neither quantitative-only nor traditional fidelity-based implementation process evaluations capture the depth and breadth of factors that inform intervention uptake. This paper describes how an ethnographic approach to process evaluation, using methods adapted to the complex world of primary care, is being used to capture the intricacies of implementing an HIT-based intervention in a manner acceptable to both clinical and research worlds. It also demonstrates how these approaches are essential to the larger mixed methods translational study.

We identified many benefits to this approach to process evaluation. As noted, the ethnographic findings are generated in a manner that is minimally disruptive to clinic workflows. In addition, the iterative nature of ethnographic data collection and analysis allows preliminary results to be shared with clinical and HIT partners as implementation unfolds, supporting mid-stream shifts in implementation strategies, as necessary. Importantly, ethnography encourages exploration of the *meaning* providers and staff attribute to the changes to care recommendations underlying the intervention, and the HIT tools through which these recommendations are implemented. This in turn provides rich insight into the complex forces underlying the success of this intervention. Much of what motivates responses to specific actions or events – such as implementation of HIT-based and other interventions – lies outside of our awareness [49]. The ethnographic data collection methods presented here let us investigate the cultural, political and environmental context in which the implementation occurred, and the connection of this context to human action [50]. Ethnography provided the framework to explore and document the evolving perspectives and actions of clinic staff, and the tools to make explicit the tangible and intangible details and relationships that influence intervention uptake. Paying attention to formal and informal intervention-related activities, and to the anticipated and unanticipated consequences of implementation, increases understanding of the ‘how’ and ‘why’ behind intervention outcomes, which in turn increases the credibility, usability and transferability of findings [23].

There are potential limitations to using ethnographic methods in process evaluations, most notably the amount of person-time required for data collection and analysis. Our team has study-funded site coordinators and substantial time devoted to the process evaluation – the lead ethnographer and assistant are budgeted at 60% and 20% full-time equivalent (fte) respectively; the four site coordinators share a total of 2.5 fte. Given research funding realities that often may not be possible. While inadequate researcher time will substantially reduce results’ explanatory power, future research might explore ways to minimize data collection time while maintaining robust findings.

Any research incorporating participant self-report must address the issue of social desirability response bias, or the tendency to respond to questions in a socially acceptable direction [51]. While some clinic staff surely softened their criticisms, omitted certain complaints and/or chose not to expose certain organizational traits when discussing the intervention with us, we believe that the ethnographic approach to data collection served to mitigate this concern. The relationships and trust we built and strengthened over the course of the study, the emphasis on data collection from researchers with both insider and outsider status, the multiple perspectives we sought, the mix of methods and data sources, and the length of time (five years) that we were in and out of the clinics allowed us to see and hear the negative as well as the positive.

Apart from person-time, additional costs are relatively minor: we give $5 coffee cards to staff who participate in the formal interviews, reimburse the clinics for provider time off the floor (20 minute interviews for 22 providers), and provide food for the group discussions. Researchers will need digital recorders that conform to their institution’s data confidentiality policies, and software for organizing the data to enable analysis (we used QSR NVivo but there are less expensive options). Transcription costs range from $35-$70 an hour; a half hour interview takes an average 1.5 hours to transcribe, a one hour group discussion approximately five hours. Transcription costs can be reduced by relying predominantly on detailed field notes and only transcribing specific key interviews and discussions.

In conclusion, we believe an ethnographic approach to process evaluation can yield the insight necessary to appropriately support cross-setting implementation of HIT-based interventions. We encourage others to share their own experiences with ethnography in implementation evaluation, and to consider adapting the methods and tools described here for their own research.

## Additional files

Additional file 1:
**A.L.L. Study - Weekly Site Coordinator Feedback Log.**
Additional file 2:
**Staff Feedback Survey.**
