# Supplementary material for: Ethnographic process evaluation in primary care: explaining the complexity of implementation
Source: BMC Health Serv Res. 2014 Dec 5;14:607. doi: 10.1186/s12913-014-0607-0 (PMC4265455; doi:10.1186/s12913-014-0607-0)
Supplement: Additional file 1: — A.L.L. Study - Weekly Site Coordinator Feedback Log. [file 12913_2014_607_MOESM1_ESM.pdf]

A.L.L. Study - Weekly Site Coordinator Feedback Log

**Date:**		**Name:**	

Please include anything you think might help us understand barriers and facilitators to OCHIN A.L.L. implementation.

Reminders:

- Goal is to reveal the stories and ongoing process of implementation.
- Please be specific and include details (how, who, what, & when) whenever possible.
- Note the feedback source (i.e., nurse, doctor, MA, patient, etc.)
- Use square brackets when sharing your insights or interpretations.
- Use quotation marks for verbatim quotes.

Potential topics for your feedback log:

- ✓ Implementation (Day-to-day logistics)
- ✓ Communication (Formal and Informal)
- ✓ Tools (BPA, Inreach and Outreach Solutions rosters, and education materials)
- ✓ Surprises, Challenges, and Solutions
- ✓ Unresolved or ongoing issues
- ✓ Other feedback that you think is relevant
