# Supplementary material for: Ethnographic process evaluation in primary care: explaining the complexity of implementation
Source: BMC Health Serv Res. 2014 Dec 5;14:607. doi: 10.1186/s12913-014-0607-0 (PMC4265455; doi:10.1186/s12913-014-0607-0)
Supplement: Additional file 2: — Staff Feedback Survey. [file 12913_2014_607_MOESM2_ESM.pdf]

Staff Feedback Survey

51281 7/12 CHR

Date:

Clinic: Clinic A Clinic B

The purpose of this survey is to inform us about how you heard about certain changes in the diabetes standard of care (sometimes called the A.L.L. Study). There are no 'right' answers, just answer as best you can.

When finished, please return the survey to:

[Name]
A.L.L. Study Coordinator

Present position **(PLEASE CHECK *ALL* THAT APPLY)**:

- Primary Care Provider
- RN
- Manager
- Patient Care Coordinator
- Team Assistant
- Medical Assistant
- Other **(PLEASE SPECIFY)**:

____

Q1) Please tell us, by checking the appropriate box under each statement, whether you knew the information below **before taking this survey**. If what you heard was different, please tell us how. **(PLEASE CHECK ONLY ONE BOX PER STATEMENT)**

a) For patients with diabetes that fit specific clinical criteria, [Org A] now recommends prescribing two medications, an ACE-inhibitor and a statin, to prevent cardiovascular events like heart attack and stroke.

- Did not know this
- Knew this
- Heard differently **(WHAT)**:

____

b) All patients with diabetes age 55-75 should be prescribed an ACE-inhibitor and a statin, subject to clinical judgment.

- Did not know this
- Knew this
- Heard differently **(WHAT)**:

____

c) Patients age 18-54 with diabetes and cardiovascular disease should be prescribed an ACE-inhibitor and a statin, subject to clinical judgment.

- Did not know this
- Knew this
- Heard differently **(WHAT)**:

____

d) These recommendations are evidence-based changes to the standard of care at [Org A], and will continue once the A.L.L. study is complete.

- Did not know this
- Knew this
- Heard differently **(WHAT)**:

____

PLEASE CONTINUE...

If you checked 'Did not know this' for every statement, then you are done.
Please return the survey to [Name], A.L.L. Study Coordinator.

If you had heard any of this information before, please continue.

Q2a) By what method(s) did you hear or read about any of this information?
(PLEASE CHECK ALL THAT APPLY)

WRITTEN COMMUNICATION

Formal email from management*
Informal email*
Notice on staff board
Best Practice Alert in EHR
Patient education materials
Other **(PLEASE SPECIFY)**:

____

VERBAL COMMUNICATION*

Attending an organized meeting*
In-person conversation*
Telephone conversation*
Overheard someone*
A.L.L. Study training*
Other **(PLEASE SPECIFY)**:*

____

b) If you heard by either email or verbal communication (*), from whom did you receive this information?

Primary Care Provider
Medical Assistant
Team Assistant
Clinic manager
Patient

RN
Patient Care Coordinator
A.L.L. Study coordinator
Senior leadership
Other **(PLEASE SPECIFY)**:

____

Q3) Where were you when you first learned about any of this information?
(PLEASE CHECK ONLY ONE BOX)

At a staff meeting (includes A.L.L. training) at this clinic
At a staff meeting at a different site
In my work area
In an exam room
Away from my work area, on a break (coffee, lunch, etc.)
Away from this clinic but still working
Away from this clinic and not working
Other **(PLEASE SPECIFY)**:

Q4) How long ago did you first receive any of this information?
(PLEASE CIRCLE ONLY ONE)

Today	1	2	3	4	5	6		Days ago
	1	2	3	4				Weeks ago
	1	2	3	4	5	6	7+	Months ago

—Thank You—
